# Modulating intracellular calcium dynamics with alkaloids: A novel strategy against oxidative neurodegeneration

**DOI:** 10.1093/toxres/tfaf100

**Published:** 2025-07-27

**Authors:** Serap NİĞDELİOĞLU DOLANBAY

**Affiliations:** Gazi University, Faculty of Science, Department of Biology, Teknikokullar, Ankara 06500, Türkiye

**Keywords:** Alkaloid-mediated calcium modulation, calcium homeostasis, calcium signalling, calcium channels, H₂O₂-induced calcium dysregulation

## Abstract

Calcium homeostasis plays a pivotal role in neuronal function, and its dysregulation is closely associated with oxidative stress-induced neurotoxicity. This study investigated the protective effects of a methanol alkaloid extract (MAE), rich in allocryptopine, tetrahydropalmatine, and tetrahydroberberine N-oxide, on H₂O₂-induced calcium dysregulation in fPC12 cells. Flow cytometry analysis revealed that MAE pretreatment significantly attenuated intracellular Ca^2+^ accumulation caused by oxidative stress. In line with this, MAE markedly downregulated the mRNA and protein expression levels of CACNA1C (Cav1.2 subunit) and CACNA1D (Cav1.3 subunit), two L-type voltage-gated calcium channels responsible for calcium influx. Furthermore, MAE suppressed the expression of key calcium regulatory proteins, including CALM1, CaMK2A, PMCA (ATP2B1), SERCA (ATP2A1), RyR1, and IP3R (ITPR1), as confirmed by ELISA and Western Blot analysis. Protein–protein interaction (PPI) network analysis demonstrated a highly interconnected and functionally enriched network among these targets, indicating coordinated regulation of calcium signaling pathways. Molecular docking studies supported these findings by showing strong binding affinities of MAE’s isoquinoline alkaloids, particularly tetrahydropalmatine, to SERCA (ATP2A1) and IP3R (ITPR1). These interactions suggest a direct modulatory effect on calcium-handling proteins. Overall, this study provides experimental and *in silico* evidence that MAE exerts multifaceted neuroprotective effects by restoring calcium homeostasis and modulating oxidative stress responses, highlighting its therapeutic potential in calcium-related neurodegenerative conditions.

## Introduction

Oxidative stress, characterized by the excessive production of reactive oxygen species (ROS), plays a pivotal role in the pathogenesis of numerous neurodegenerative diseases.^1^^,^^2^ Hydrogen peroxide (H₂O₂), a stable and permeable ROS, is widely used as a model compound to induce oxidative damage in *in vitro* systems, offering valuable insights into cellular defense mechanisms.^3^^,^^4^ Among the most important results of oxidative stress is impaired intracellular calcium handling, which is tightly associated with critical cellular functions such as neurotransmission, enzyme activity, and gene expression.^5^^,^^6^ In normal physiology, calcium is tightly controlled within a certain level that is maintained by a strong interaction between calcium entry, release, and sequestration through cellular membrane, endoplasmic reticulum, and mitochondria.^7^^,^^8^ Nevertheless, when ROS such as hydrogen peroxide reach excessive amounts within a cell, they lead to disruption of calcium channels and pumps, resulting in calcium accumulation in excessive amounts within a cell.^9^^,^^10^ This causes mitochondrial dysfunction as well as further ROS generation; furthermore, it can lead to cell death by starting a chain of signaling events.^11^^,^^12^

Particularly calcium signaling includes voltage-gated calcium channels such as CACNA1C (Cav1.2 subunit) and CACNA1D (Cav1.3 subunit) as well as a closely related web of regulatory proteins.^13^^,^^14^ This system’s critical components include calmodulin (CALM) with primary calcium sensor properties, and calcium/calmodulin-dependent protein kinase II (CaMKII) converting calcium signals into functional responses exerted by cells.^15^^,^^16^ Plasma membrane calcium ATPases (PMCA; ATP2B1), sarco/endoplasmic reticulum calcium ATPases (SERCA; ATP2A2), and calcium release channels including ryanodine receptors (RyR) and inositol 1,4,5-trisphosphate receptors (IP3R) are responsible for the removal and storage of calcium.^17^^,^^18^ When dysfunctional, any member of this system can worsen mitochondrial dysfunction, oxidative stress, and cell death.^19^^,^^20^

Plant-derived alkaloids have recently been a focus of scientific attention owing to their strong antioxidant, anti-inflammatory, and neuroprotective properties.^21^^,^^22^  *Glaucium grandiflorum* Boiss. & A. Huet subsp. *refractum* (Nábělek) Mory is a member of the Papaveraceae family and contains a considerable amount of natural isoquinoline alkaloids.^23–26^ The latter both modulate oxidative stress and inflammation, and potentially regulate intracellular calcium balance. When impaired, calcium homeostasis causes mitochondrial dysfunction and cellular demise of neuronal cells. Thus, it is of paramount importance to discover the mechanisms with which alkaloids originating from *Glaucium grandiflorum* affect calcium signaling in order to have a decent understanding of how plants exert neuroprotection on a molecular level.

We previously determined the presence of main alkaloids (allocryptopine, tetrahydropalmatine, and tetrahydroberberine N-oxide (trans-cannadine-N-oxide) in the *G. grandiflorum* alkaloid extract with the help of GC–MS, LC–MS/MS, AND _1_H, _13_C NMR analyses.^23^^,^^24^ We previously showed that the alkaloid extract of *G. grandiflorum* with high allocryptopine, tetrahydropalmatine, and tetrahydroberberine N-oxide content reduced the rate of neuronal apoptosis caused by oxidative stress (H_2_O_2_), which may involve the suppression of the mitochondrial apoptotic pathway and regulation of the cell cycle in PC12 cells.^23^ According to our results on a previous study on BV2 microglial cells, the alkaloid extract obtained from *G. grandiflorum* mitigated inflammation caused by lipopolysaccharide (LPS) by inhibiting the pro-inflammatory cytokines, mediators, and the p38 MAPK pathway.^24^ We also previously demonstrated that the regulatory effects on the NRF2-KEAP1 in PC12 cells exerted by the alkaloid extract of *G. grandiflorum,* which contains high amounts of allocryptopine, tetrahydropalmatine, and tetrahydroberberine N-oxide, reduced H_2_O_2_ -induced oxidative stress.^25^

Although, we showed that the alkaloid extract produced by *Glaucium grandiflorum* subsp. *refractum* exerted antioxidant, anti-apoptotic, and anti-inflammatory activity in several studies, it is still unclear how these activities are related to intracellular calcium signaling on a molecular level. We thus meticulously scrutinized how the methanol alkaloid extract (MAE) regulates calcium balance for the first time in the literature. The present study aims to investigate this mechanism in detail in an attempt to explain the previous observations hinting at a protective activity of MAE on cells in a mechanistic way so that the therapeutic value of the extract for the treatment of oxidative stress-induced neuronal dysfunction can be better delineated.

## Materials and methods

### Plant material and methanol alkaloid extract (MAE) preparation

The present study employed *Glaucium grandiflorum* Boiss. & A. Huet subsp. *refractum* (Nábělek) Mory, which was collected from Beypazarı (Ankara, Turkey) on 2015 July 27 by Prof. Dr Zeki Aytaç and stored at the Gazi University Herbarium (voucher no: ZA10700). The aerial portions of the plant were dried, powdered, and stored for use in the experiments.

Methanol was used to extract 10 gr powdered plant material with the use of a Soxhlet apparatus (LabHeat) for a period of 8 hours. Then, solvent was removed by rotary evaporation (40 °C) (Heidolph Laborota), followed by the extract’s acidification, irrigation with diethyl ether, basification, and re-extraction with methanol. The last methanol phase was dried and evaporated. The MAE obtained by the above-mentioned steps was then deposited at +4 °C until further use in the experiments.

### Cell treatments

This study used the pheochromocytoma-12 (PC12, ATCC® CRL-1721™) cell line obtained from the adrenal medulla of *Rattus norvegicus*. Cells were subjected to culturing in DMEM (Gibco) with additional 10% heat-inactivated fetal bovine serum (Gibco), 10% horse serum (Gibco), 1% penicillin/streptomycin (Sigma-Aldrich), and 1% L-glutamine (Gibco) in a humidified 5% CO₂ incubator with a culture temperature of 37 °C (Panasonic).

PC12 cell growth was achieved using flasks (Corning) with collagen coating (Thermo Fisher Scientific), and media were replenished every other day. A confluency of 80–90%, when reached, marked the time for cells to be seeded into plates containing 96 or 6 wells (10^4^–5 × 10^5^ cells/well), depending on the requirements of the experiment. NGF (Promega) at a concentration of 100 ng/ml was used to facilitate neuronal differentiation for a period of 4 days. In accordance with the previously published optimization studies based on MTT,^23^ four groups were formed for the experiments:

*Control:* Cells were subjected to culturing in DMEM with added NGF for a period of 6 days and no additional treatment was applied.

*H₂O₂ (Neurodegenerative model):* On the 6th day of the experiment, fPC12 cells were subjected to treatment with 200 μm H₂O₂ for a period of 24 hours.

*MAE:* On the 5th day of the experiment, fPC12 cells were subjected to treatment with MAE with a concentration of 100–500 μg/ml for a duration of 18 hours.

*MAE + H₂O₂:* A pre-treatment with MAE at a concentration of 100–500 μg/ml was applied to cells for a period of 18 hours; they were subsequently subjected to treatment with 200 μm H₂O₂ for a period of 24 hours to evaluate neuroprotection.

### Flow cytometry analysis

PC12 cells were seeded on plates that were coated with collagen and containing 6 wells (1 × 10^5^ cells/well). fPC12 cells were then treated with NGF at a concentration of 100 ng/ml for a duration of 4 days and left to an 18-hour incubation using MAE at concentrations of 100, 250, and 500 μg/ml. After that step, cells were subjected to treatment with 200 μm H₂O₂ in a medium containing NGF for a duration of 24 hours. The control group was formed by cells that were kept in the NGF medium alone. The treatment of the cells was followed by their harvest, centrifugation (at a rate of 1,000 rpm for a period of 10 minutes), and resuspension in PBS containing no calcium. The cells were stained using 5 μm eFluor™ 514 (Invitrogen) at a temperature of 37 °C for a period of 30 minutes in the dark, and then at room temperature for a duration of 15 minutes. After washing wuth PBAs twice, the intensity of fluorescence was quantified (excitation: 490 nm, emission: 514 nm) and recorded as times change in relation to the control value. Flow cytometry with NovoCyte® 3,000 and 2060R instruments (ACEA Biosciences and Agilent) was followed by data analysis using the ACEA NovoExpress software. THORVACS Biotechnology Laboratory at Bilkent University was used for conducting all analyses.

### qRT-PCR analysis

Commercial kits (Qiagen, Zymo Research, Invitrogen) used in compliance with the manufacturer’s guides were used for total RNA isolation from fPC12 cells. SensiFAST cDNA Synthesis Kit (Bioline) and High-Capacity cDNA Reverse Transcription Kit (Thermo Fisher Scientific) were used to synthesize cDNA. qRT-PCR was conducted using primers that were specific for CACNA1C (Cav1.2), CACNA1D (Cav1.3), and the housekeeping gene GAPDH; the kits used for qRT-PCR were the SensiFAST™ SYBR® Lo-ROX Kit (Bioline) and AMPIGENE® Green Mix (Enzo) and the platform for qRT-PCR was a QuantStudio™ 3 Real-Time PCR System (Thermo Fisher Scientific). The following thermal cycling conditions were used: 95 °C for 2 minutes (activation), followed by 40 cycles of 95 °C for 5 seconds (denaturation), 60–65 °C for 10–30 seconds (annealing), and 72 °C for 20 seconds (extension). Gene expression level normalization was done to GAPDH and the levels were analyzed with the 2^–∆∆CT^ method in comparison with the control group.

### Western blot analysis

Treated and untreated cells were washed with cold PBS and centrifuged at 2,500 × g for 5 minutes at +4 °C. Protease and phosphatase inhibitor tablets were added to the cell pellets, followed by the addition of 500 μL of cold RIPA lysis buffer. The samples were incubated for 30 minutes at +4 °C with vortexing every 5 minutes. After incubation, the tubes were centrifuged at 16,000 × g for 30 minutes at +4 °C. The protein content in the supernatant was quantified using the BCA Protein Assay Kit. A standard curve was generated using BSA standards ranging from 25 to 2000 μg/ml, and absorbance was measured at 562 nm to determine the protein concentration in the samples. A 1:1 mixture of the protein samples (10–30 μg) and 2× Laemmli buffer (5% β-mercaptoethanol) (Biorad) was formed and left to denaturation at a temperature of 90 °C for a duration of 10 minutes. Sample separation was accomplished on 4–20% SDS-PAGE gels (Bio-Rad) at 120 V for a duration 60 minutes, followed by the transfer of the samples to PVDF membranes with the help of the Trans-Blot Turbo System (Biorad). Membrane block was performed in 5% non-fat milk in 1× TBS (Biorad) with 0.1% Tween-20 for a duration of 1 hour, followed by their irrigation (3 × 5 min). The samples were then treated with primary antibodies for a period of 2 hours, as well as secondary antibodies for a duration of 1 hour (Table 1). After the samples were washed, an AP substrate kit (Bio-Rad) was utilized for detection. Membranes were then washed out using distilled water for removal of excess substrate. Visualization of protein bands was performed with the Gel Doc™ XR+ system (Biorad), and densitometric analysis was performed using the Image Lab 6.0.1 software (Bio-Rad).

**Table 1:** Primary and seconder antibodies used for western blot, dilution ratios, molecular weights and manufacturers.

**Antibody name**	**Catalog number**	**Dilution ratio**	**Molecular weight** **(kDa)**	**Manufacturer** **company**
GAPDH	10,494-1AP	1/2000	36	Proteintech
CACNA1C	orb335100	1/200	249	Biorbyt
CACNA1D	PA5-78902	1/10-5/10	245	Thermo Fisher Scientific
CALM1	ab61001	1/500	17	Abcam
CaMK2A	sc-13,141	1/100	50	Santa Cruz
PMCA (ATP2B1)	Ab190355	1/1000	135	Abcam
SERCA (ATP2A1)	#12293	1/1000	100	Cell Signaling Technologies
RyR1	26,968-1-AP	1/500	565	Proteintech
IP3R (ITPR1)	ab264281	1/2000	313	Abcam
Goat anti-rabbit IgG H + L), AP conjugate	SA00002-2	1/1000	–	Proteintech
Goat anti-mouse IgG (H + L), AP conjugate	31,320	1/5000	–	Thermo Fisher Scientific

### ELISA analysis

The levels of CALM1, CaMK2A, PMCA (ATP2B1), SERCA (ATP2A1), RyR1, and IP3R (ITPR1) were measured in accordance with manufacturer’s specifications provided for the purchased kit (Abbexa, Cusabio, MyBioSource, and ELK Biotechnology).

### Protein–protein interaction (PPI) analysis

Protein–protein interaction (PPI) networks were created to scrutinize any potential interaction between proteins involved in calcium signaling, which included CALM1, CaMK2A, PMCA (ATP2B1), SERCA (ATP2A1), RyR1, and IP3R (ITPR1). The STRING database (v11.5) [https://string-db.org] was used to perform the analysis; in that database, both established and estimated protein–protein associations extrapolated from experimental data, computational estimation methods, and public text collections were compiled.

### Molecular docking analysis

Three alkaloids found in MAE—allocryptopine, tetrahydropalmatine, and tetrahydroberberine N-oxide—were downloaded from the PubChem site in .sdf format. Target proteins [CALM1, CaMK2A, PMCA (ATP2B1), SERCA (ATP2A1), RyR1, and IP3R (ITPR1)] were retrieved as pdb format from the RCSB Protein Data Bank. Blind docking was performed with the help of CB-Dock2 and based on cavity detection.

### Statistical analysis

Statistical analyses were carried out with SPSS software package. Inter-group differences were tested using independent t-test or one-way ANOVA test, with Tukey’s HSD as the post hoc test. Data are shown in the form of mean ± SD from parallel replicates. *P* values of less than 0.05 and 0.01 were considered statistically significant.

## Results

### Effects of MAE on H₂O₂-induced intracellular Ca^2+^ levels in fPC12 cells

**Fig. 1:**
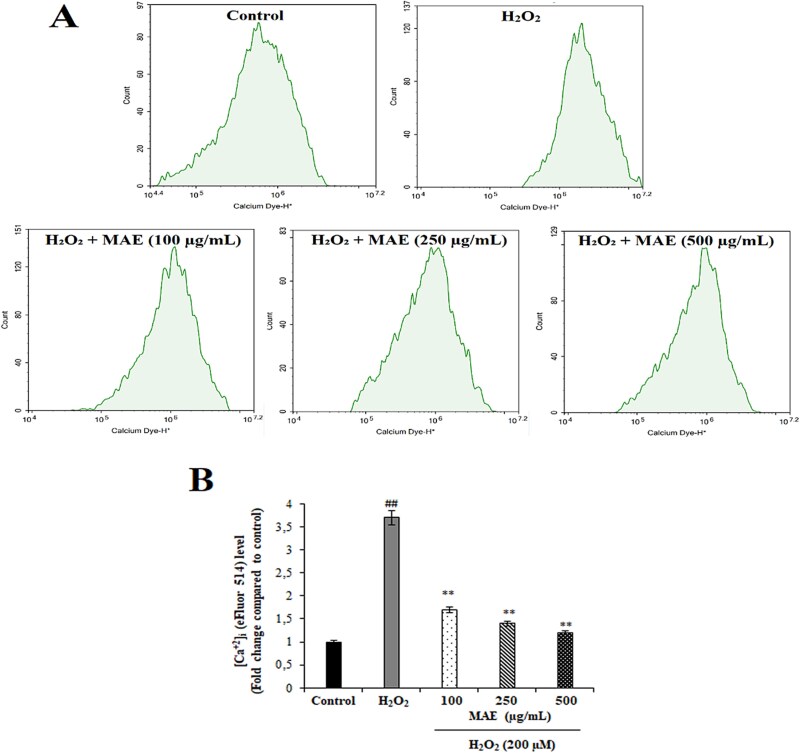
Suppressive effects of MAE on H_₂_O_₂_-induced intracellular Ca^2+^ levels measured by flow cytometry. A: Representative flow cytometry histogram showing [Ca^2+^]i distribution based on eFluor 514 fluorescence. B: Quantitative analysis of intracellular Ca^2+^ levels presented as fold change relative to the control group. Data are expressed as mean ± SD (n = 3). ^##^*P* < 0.01 vs. control group; ^**^P < 0.01 vs. H_₂_O_₂_-treated group.

The effect of MAE on H₂O₂-induced intracellular calcium levels was determined by analyzing fPC12 cells with flow cytometry using eFluor 514, a fluorescent calcium indicator. As compared with the controls, the H₂O₂-treated group showed a significant rightward shift in fluorescence intensity in flow cytometric histograms, which was a marker of marked intracellular Ca^2+^ increase (^##^*P* < 0.01). Intracellular [Ca^2+^]i increase was significantly reduced by a pretreatment with MAE, which was indicated by reduced intensity of fluorescence and a parallel reduction in calcium levels in comparison to the group treated solely with H₂O₂ (^**^P < 0.01). Furthermore, there was an increase in this effect with increasing concentrations of MAE, with calcium increase being reduced parallelly. MAE was also verified to reduce calcium influx occurring as a consequence of oxidative stress significantly; this effect corroborates its promising role in balancing intracellular calcium dynamics in face of neurotoxicity (Fig. 1).

### Effects of MAE on H₂O₂-induced CACNA1C (Cav1.2 subunit) and CACNA1D (Cav1.3 subunit) gene expression in fPC12 cells

**Fig. 2:**
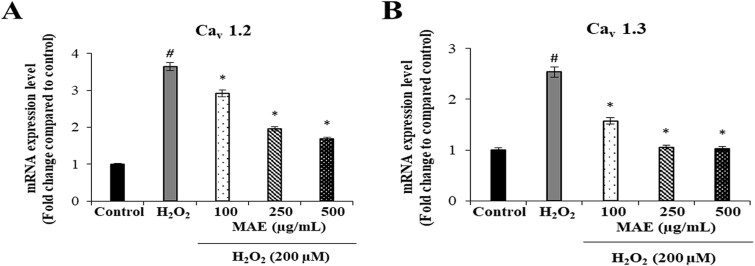
Suppressive effects of MAE on H_₂_O_₂_-induced CACNA1C (Cav1.2 subunit) and CACNA1D (Cav1.3 subunit) mRNA expression levels in fPC12 cells. A: Cav1.2; B: Cav1.3 gene expression levels in fPC12 cells pretreated with MAE, following L-type VGCC activation by H_₂_O_₂_. Data are expressed as mean ± SD (n = 3). ^#^*P* < 0.05 vs. control group; ^*^P < 0.05 vs. H₂O₂-treated group.

The regulatory effects of MAE on L-type voltage-gated calcium channel (VGCC) subtypes were studied by assessing the CACNA1C (Cav1.2 subunit) and CACNA1D (Cav1.3 subunit) genes’ expression levels in fPC12 cells in the case of H₂O₂ induced oxidative stress. According to the qRT-PCR analysis, H₂O₂ significantly caused the mRNA expression levels of both CACNA1C (Cav1.2 subunit) and CACNA1D (Cav1.3 subunit) to significantly increase than those of the control group (^#^*P* < 0.05), which suggested that oxidative stress promotes the expression of L-type VGCC gene. Pretreatment with MAE significantly reduced the upregulation of both CACNA1C (Cav1.2 subunit) and CACNA1D (Cav1.3 subunit) genes brought about by H₂O₂ (^*^P < 0.05). Furthermore, there was an increase in this inhibitory effect on gene expression with increasing concentrations of MAE. According to these results, it was evident that MAE has an inhibitory activity on the expression of L-type VGCC subunit when oxidative stress is in place, and it provides protection for neuronal cells against cytotoxic effects of calcium inflow by limiting the amount of the latter (Fig. 2).

**Fig. 3:**
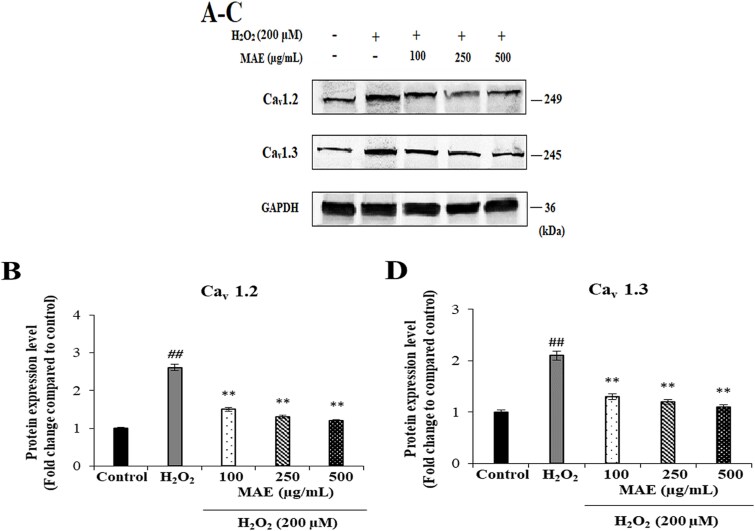
Suppressive effects of MAE on H_₂_O_₂_-induced CACNA1C (Cav1.2 subunit) and CACNA1D (Cav1.3 subunit) protein expression in fPC12 cells. A: Representative immunoblot and quantitative analysis of CACNA1C (Cav1.2 subunit) protein levels. B: Representative immunoblot and quantitative analysis of CACNA1D (Cav1.3 subunit) protein levels. Data are expressed as mean ± SD (n = 2). ^##^*P* < 0.01 vs. control group; ^**^P < 0.01 vs. H₂O₂-treated group.

### Effects of MAE on H₂O₂-induced CACNA1C (Cav1.2 subunit) and CACNA1D (Cav1.3 subunit) protein expression in fPC12 cells

The impact of MAE on L-type VGCC protein expression in the case of oxidative stress was evaluated by measuring CACNA1C (Cav1.2 subunit) and CACNA1D (Cav1.3 subunit) protein levels using Western blot analysis in fPC12 cells after their treatment with H₂O₂. The expression levels of both CACNA1C (Cav1.2 subunit) and CACNA1D (Cav1.3 subunit) proteins (^##^*P* < 0.01) were significantly upregulated by H₂O₂, which pointed to the phenomenon of the upregulation of these VGCC subtypes under oxidative stress. MAE, when used for pretreatment, significantly reduced the levels of the CACNA1C (Cav1.2 subunit) and CACNA1D (Cav1.3 subunit) proteins that rose in response to H₂O₂ treatment (^**^P < 0.01). It was also evident that the reductory effect of MAE on protein expression levels increased in parallel to its increasing concentrations. The above finding indicates that the expression of L-type VGCC was reduced by MAE in the presence of oxidative stress, a feature of MAE that may be involved in its neuroprotective role played by suppressing cell toxicity caused by calcium inflow (Fig. 3).

**Fig. 4:**
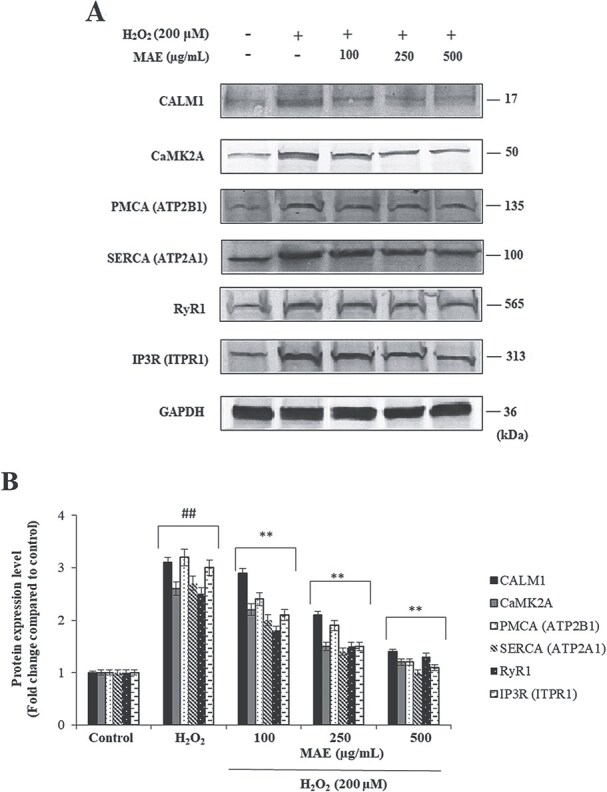
Suppressive effects of MAE on H_₂_O_₂_-induced CALM1, CaMK2A, PMCA (ATP2B1), SERCA (ATP2A1), RyR1, and IP3R (ITPR1) protein expression in fPC12 cells. A: Representative immunoblot and quantitative analysis of protein levels. B: Representative immunoblot and quantitative analysis of protein levels. Data are expressed as mean ± SD (n = 2). ^##^*P* < 0.01 vs. control group; ^**^P < 0.01 vs. H₂O₂-treated group.

### Effects of MAE on H₂O₂-induced expression of calcium regulatory proteins in fPC12 cells

ELISA and Western Blot analyses were conducted in H₂O₂-treated fPC12 cells in an attempt to study how MAE would affect the expression of key proteins involved in intracellular calcium handling in the presence of oxidative stress. The protein levels of CALM1, CaMK2A, PMCA (ATP2B1), SERCA (ATP2A1), RyR1, and IP3R (ITPR1) increased significantly in response to treatment with H₂O₂ as compared with the control group (^#^*P* < 0.01), suggesting a disruptive effect of oxidative stress on calcium handling via changing the expression of the above-mentioned vital regulatory proteins. The levels of those proteins did not elevate in response to H₂O₂ when the medium was pretreated with MAE (^*^P < 0.01). Besides, the effect of MAE on protein levels was strengthened by its increasing concentrations. This finding is indicative of the fact that, by altering the expression levels of the proteins involved in calcium homeostasis in a concentration-dependent manner, MAE is able to revert the intracellular calcium equilibrium back to normal in the presence of oxidative stress. This also corroborates the beneficial effects of MAE in the normalization of calcium signaling and its promising role in the field of neuroprotection (Table 2 and Fig. 4). The ELISA and Western Blot results are consistent and support each other. Both methods show similar trends, with increased protein expression following H₂O₂ treatment and a dose-dependent decrease upon MAE administration. This parallel pattern indicates that the findings from both assays are in agreement and reinforce the reliability of the observed effects.

**Table 2:** Suppressive effects of MAE on H₂O₂-induced expression levels of intracellular calcium regulatory proteins in fPC12 cells.

**Treatment**	**Relative protein expression levels**					
	**CALM1**	**CaMK2A**	**PMCA** **(ATP2B1)**	**SERCA** **(ATP2A1)**	**RyR1**	**IP3R** **(ITPR1)**
Control	1.0 ± 0.3	1.0 ± 0.1	1.0 ± 0.2	1.0 ± 0.0	1.0 ± 0.2	1.0 ± 0.1
200 μm H_2_O_2_	3.2 ± 0.1^#^	2.7 ± 0.3^#^	3.1 ± 0.2^#^	2.8 ± 0.3^#^	2.6 ± 0.1^#^	2.9 ± 0.2^#^
100 μg/ml MAE + H_2_O_2_	2.8 ± 0.2^*^	2.3 ± 0.1^*^	2.5 ± 0.3^*^	2.1 ± 0.1^*^	1.9 ± 0.2^*^	2.0 ± 0.1^*^
250 μg/ml MAE + H_2_O_2_	2.2 ± 0.2^*^	1.6 ± 0.2^*^	2.0 ± 0.3^*^	1.3 ± 0.1^*^	1.4 ± 0.3^*^	1.4 ± 0.2^*^
500 μg/ml MAE + H_2_O_2_	1.5 ± 0.3^*^	1.1 ± 0.2^*^	1.3 ± 0.2^*^	0.9 ± 0.2^*^	1.2 ± 0.2^*^	1.0 ± 0.3^*^

Quantitative ELISA analysis of CALM1, CaMK2A, PMCA (ATP2B1), SERCA (ATP2A1), RyR1, and IP3R (ITPR1) protein levels. fPC12 cells were pretreated with MAE prior to H₂O₂-induced oxidative stress. Data are expressed as mean ± SD (n = 3). ^#^*P* < 0.01 vs. control group; ^*^P < 0.01 vs. H₂O₂-treated group.

### Predicted PPI network of calcium homeostasis-associated proteins affected by MAE

**Fig. 5:**
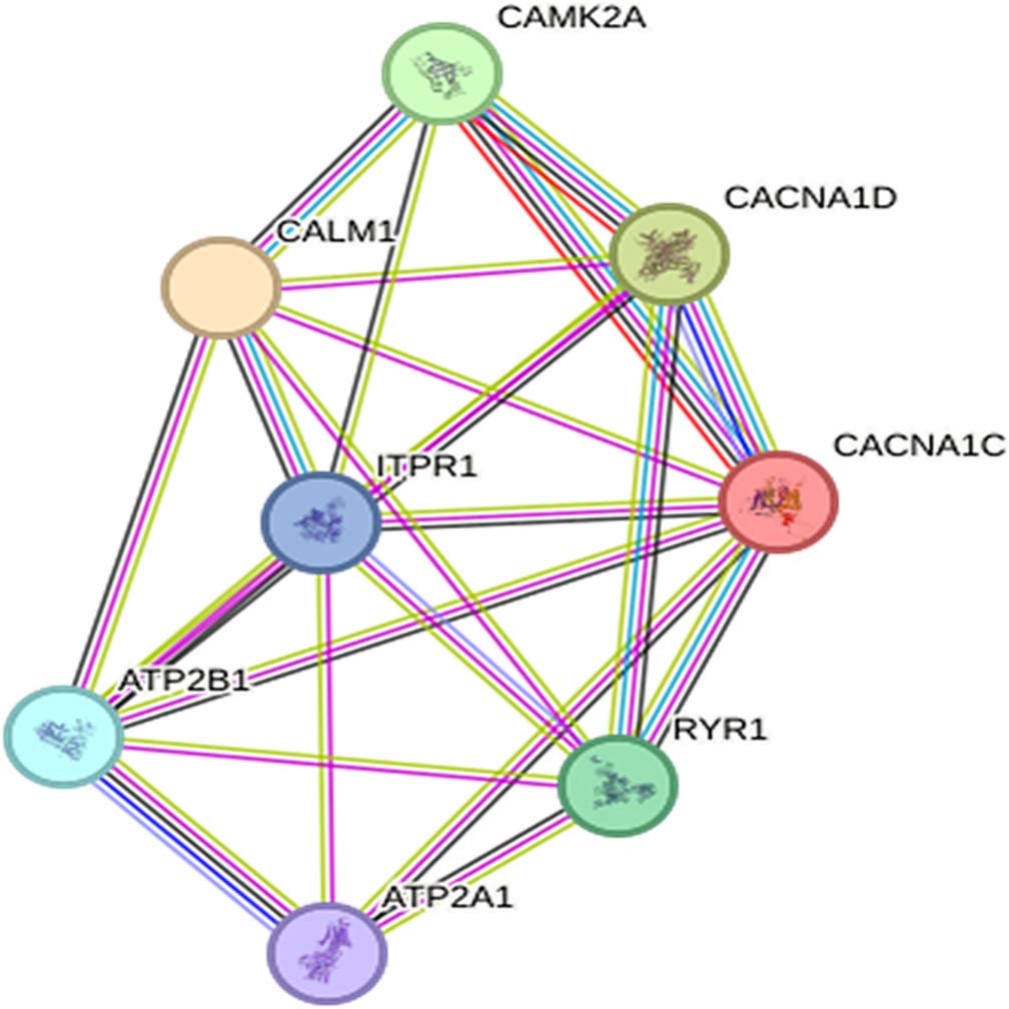
The PPI network of calcium homeostasis-associated proteins affected by MAE.

Herein, the possible interplay between proteins that are involved in calcium homeostasis and affected by MAE was investigated by creating a PPI network (Fig. 5). This network was formed using 8 proteins involved in calcium signaling and composed of 8 nodes and 23 edges. There are an average of 5.75 interactions per protein with other proteins, making the PPI structure a considerably intricate web of interconnections. An aveage local clustering coefficient of 0.874 calculated for the structure indicated that the proteins involved in it are inclined to create densely packed clusters in which there were also interconnections between the partners of interaction. Such a high clustering coefficient is a marker of a network hosting functionality interconnected modules. Although one would predict that a similarly sized random network contains only 1 edge, the actual network had 23 edges, which suggested an organization that occurred non-randomly and had a biological significance. A PPI enrichment *P*-value of <1.0e-16 also corroborates the statistical significance of the observed interplay between the components and refutes the chance factor, further signaling the presence of a robust functional connection between the proteins. The visual depiction of the network marks the levels of interaction by node colors and the type of interaction evidence by edge colors. The edge colors were used in the following manner: green indicates genomic neighborhood; red indicates gene fusion; blue indicates functional association; dark indicates co-expression; pink indicates experimental evidence; sky blue indicates curated database information; kelly green indicates text mining; and purple indicates homology. These results reveal a tightly associated, substantially congregated, and functionally enhanced interaction network between the proteins involved in calcium signaling, emphasizing their synchronized roles in the pathways of calcium signaling.

### Docking-based prediction of interactions between MAE alkaloids and key calcium signaling proteins

This study employed molecular docking analysis to determine how three major alkaloids found in the structure of MAE—allocryptopine, tetrahydropalmatine, and tetrahydroberberine N-oxide, would interact with key proteins taking part in calcium signaling pathways. Table 3 provides a detailed description of the binding energies (vina scores) and interacting amino acid residues between the ligands and their corresponding protein targets. The molecular docking analyses used in the study demonstrated that the target proteins showed ligand affinity in the following decreasing order: SERCA (ATP2A1) > IP3R (ITPR1) > CamK2A > PMCA (ATP2B1) > RyR1 > CALM1. Accordingly, SERCA (ATP2A1) displays the highest binding affinity towards the ligands whereas CALM1 displays the weakest affinity. The ligands displayed binding affinity towards the target proteins in the following decrasing order: tetrahydropalmatine > allocryptopine > tetrahydroberberine N-oxide. These results reveal that the strongest binding affinity among all proteins involved was exhibited towards tetrahydropalmatine. In general, all ligands evaluated by the molecular docking analyses displayed significant and powerful interplay with respective target proteins; this indicates a potential for biological function. Low, i.e. more negative, binding energies onserved in the analyses suggest that the ligands bind to target proteins’ active sites with hogh affinity and thus a high likelihood of target-specific and firm interactions. These findings signal a likely regulatory influence of the ligands on the target proteins involved in calcium signaling pathways.

**Table 3:** Predicted binding energies and contact residues of ligands with calcium regulatory proteins.

**Ligands**	**Target Proteins**	**Vina Scores**	**Contact Residues**	
Allocryptopine	CALM1	−6.3	Chain A: ARG86 GLU87 PHE89 ARG90 ASP93 GLY96 ASN97 GLY98 TYR99 ASP131 ASN137 TYR138 GLU139	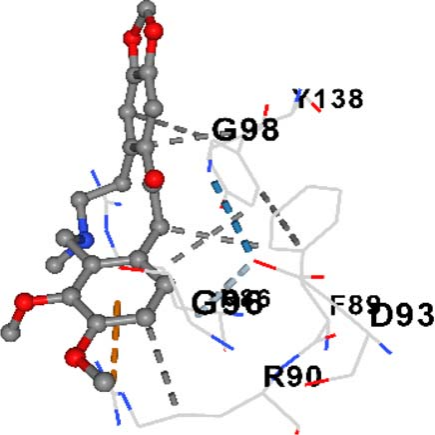
	CamK2A	−9.0	Chain A: LEU19 GLY20 LYS21 GLY22 SER25 VAL27 MET42 ILE44 LYS47 LYS48 LEU49 ASP53 HIS54 LYS56 LEU57 GLU60 CYS64 VAL73 LEU87 PHE89 ASP90 VAL92 GLY95 GLU96 ASN135 LYS137 GLU139 ASN140 LEU142 LEU154 ALA155 ASP156 PHE157 GLY158 LEU159 LYS378 PRO379 VAL380	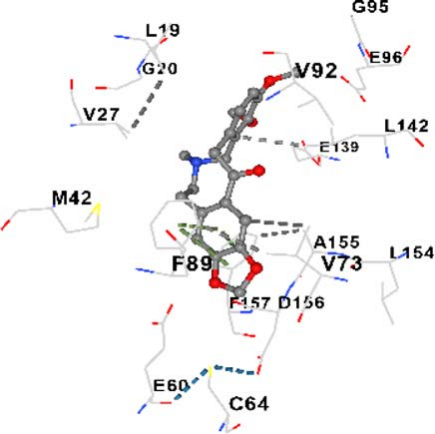
	PMCA (ATP2B1)	−7.3	Chain A: THR477 LYS583 SER584 MET585 GLY602 ALA603 SER604 GLU605 ILE606 LYS609 ARG629 VAL633 LYS634 ILE637 GLU638 MET640 GLU643 GLY644 LEU645 ARG646 THR647 ILE648 ILE682 GLY709 ASP710 ASN711 ILE712 ASN713 THR714 ARG716 GLU735 ARG767	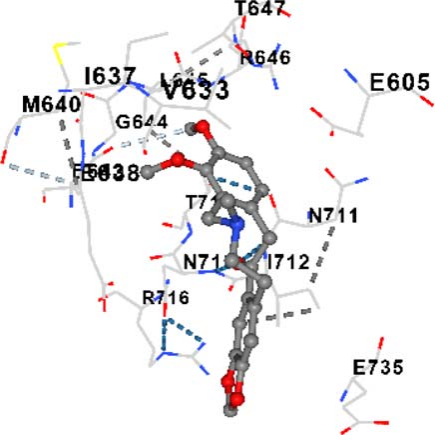
	SERCA (ATP2A1)	−8.1	Chain A: LEU52 GLU55 GLN56 PHE57 GLU58 ASP59 LEU60 VAL62 LEU98 ASN101 ALA102 VAL104 GLY105 VAL106 GLN108 GLU109 ARG110 GLU113 GLN250 LEU253 ASP254 GLY257 GLU258 LEU260 SER261 VAL304 ILE307 PRO308 GLU309 GLY310 LEU311 PRO312 ALA313 VAL314 ILE315 PHE760 LEU764 ILE765 ASP800 THR805	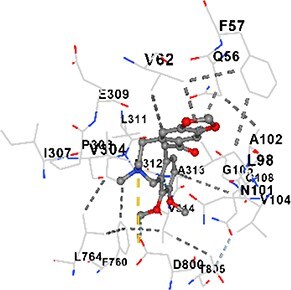
	RyR1	−6.6	Chain A: THR2762 LYS2765 TRP2766 ASP2769 HIS2788 MET2790 GLU2803 ILE2804 TYR2805 TRP2807 PRO2808 TYR2882 HIS2883 THR2885 TRP2886 LYS2889 ARG2918 ASP2919 GLU2921 LYS2922 GLU2925	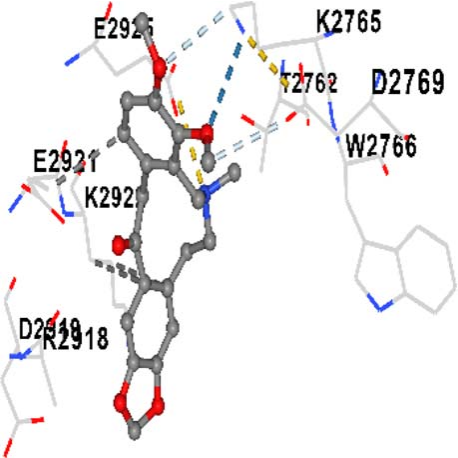
	IP3R (ITPR1)	−9.0	Chain B: ILE13 THR28 GLY30 PRO48 PRO49 LYS50 LYS51 PHE52 ARG53 LEU56 MET127 LYS128 SER129 PHE224 MET225 HIS230 LYS236 GLY237 GLY238 ASP239 VAL240 CYS254 ASP255 LEU261 GLU284 GLU286 VAL288 HIS289 PRO292 CYS293 ARG294 ARG305 LYS307 LEU309 ALA310 THR311 GLY312 ASN313 TYR314 GLY360 ASN361 ARG383 ASP443	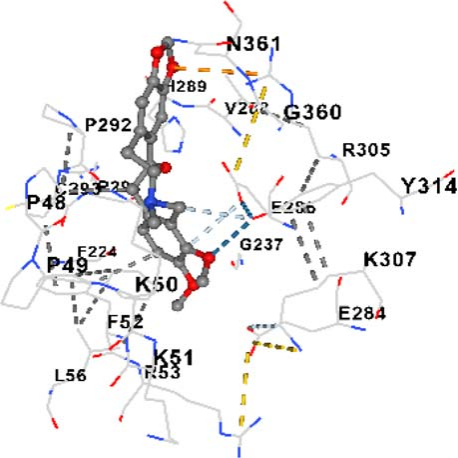
Tetrahydropalmatine	CALM1	−5.9	Chain A: ARG86 GLU87 ALA88 PHE89 ARG90 VAL91 ASP93 LYS94 ASP95 GLY96 ASN97 GLY98 TYR99 ASN137 TYR138 GLU139	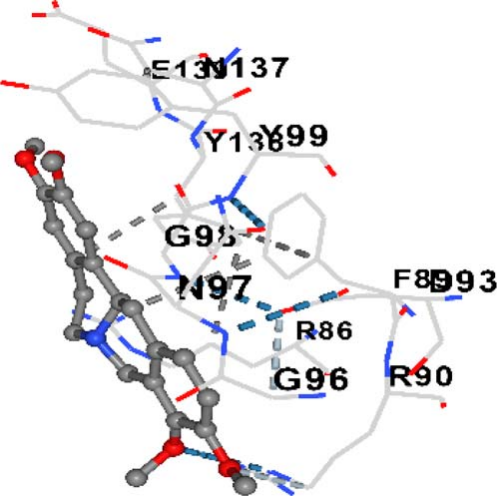
	CamK2A	−8.4	Chain A: GLY22 SER25 ASN45 THR46 LYS47 LYS48 LEU49 SER50 ASP53 HIS54 GLY83 ILE331 SER332 ASN333 TRP373 SER374 ARG375 ASN376 SER377 LYS378 PRO379 VAL380 HIS381 THR382 THR405 TYR407 LEU408 ASP409 ALA410 ALA417 ALA442 PRO443 SER444	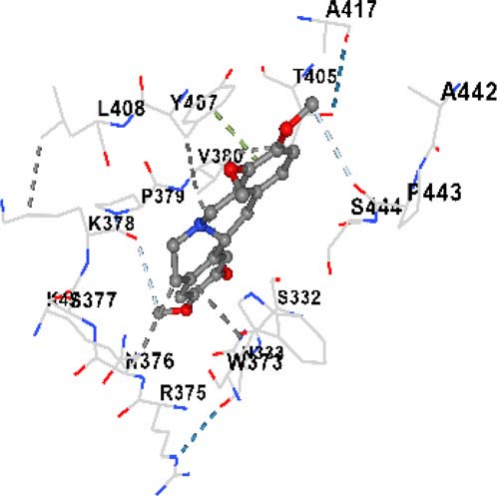
	PMCA (ATP2B1)	−8.5	Chain A: ASN847 ASP850 SER851 LYS854 GLU905 PRO906 PRO907 THR908 SER910 LEU911 LEU913 ARG914 ARG991 LYS992 ILE993 HIS994 GLY995 GLU1001 GLY1002 ASN1005	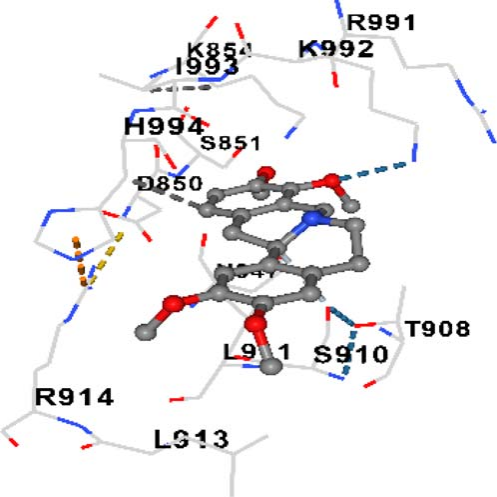
	SERCA (ATP2A1)	−9.6	Chain A: LEU52 GLU55 GLN56 PHE57 GLU58 ASP59 LEU61 VAL62 LEU98 ASN101 ALA102 VAL104 GLY105 VAL106 GLN108 GLU109 ARG110 GLU113 ALA303 VAL304 ALA305 ILE307 PRO308 GLU309 GLY310 LEU311 PRO312 ALA313 VAL314 PHE760 LEU764 ASN796 ASP800 ALA804 THR805 Chain B: ASN34	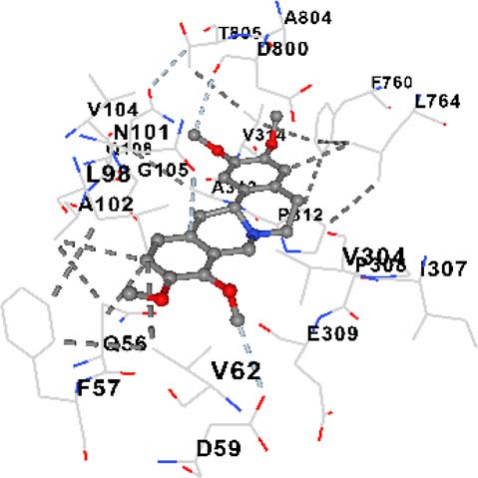
	RyR1	−6.3	Chain A: SER2753 PHE2754 LYS2757 GLU2760 TYR2761 GLU2764 LYS2825 ARG2827 GLU2828 GLY2829 GLU2830 ASN2856 PRO2857 GLN2858 PRO2859 PRO2860 ASP2861 GLY2864 VAL2865 THR2866 PHE2929 GLN2931 MET2932 ASN2933 GLY2934 TYR2935	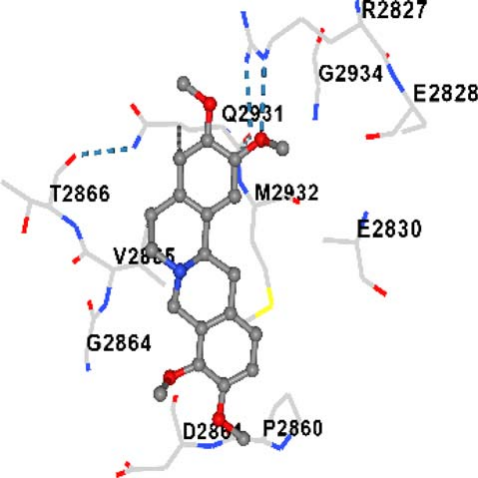
	IP3R (ITPR1)	−8.9	Chain B: GLY30 LEU31 VAL32 ASP33 ASP46 PRO48 PRO49 LYS50 LYS51 PHE52 ARG53 LEU56 LYS128 SER129 ASN130 LYS131 PHE224 GLY237 GLY238 ASP255 GLU256 LEU261 ASN280 GLU284 GLU286 VAL287 VAL288 HIS289 ASP291 PRO292 CYS293 ARG294 GLY295 ARG305 LYS307 HIS308 LEU309 ALA310 THR311 GLY312 ASN313 TYR314 PRO358 HIS359 GLY360 ASN361 ARG440 ASP441 ASP443 PHE444 ASN446 ASP447 SER449 SER450	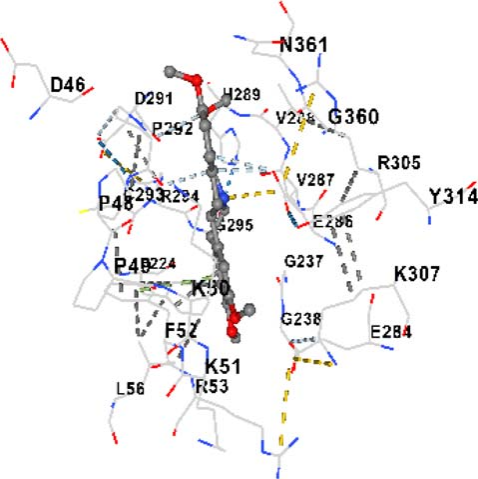
Tetrahydroberberine N-oxide	CALM1	−6.2	Chain A: ARG86 PHE89 ARG90 ASP93 GLY96 ASN97 GLY98 TYR99 ASN137 TYR138 GLU139	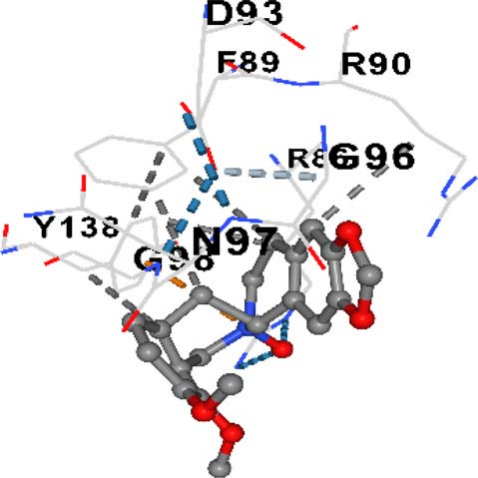
	CamK2A	−8.2	Chain A: LYS137 GLU139 THR176 PRO177 GLY178 TYR179 VAL201 ILE205 PRO211 PHE213 TRP214 ASP215 GLU216 ASP217 GLN218 LEU221 PHE293 ARG296 ARG297 LYS300 GLY301 ALA302 ILE303 LEU304 VAL306 LEU308 THR310 ASN312 GLN318 ILE321	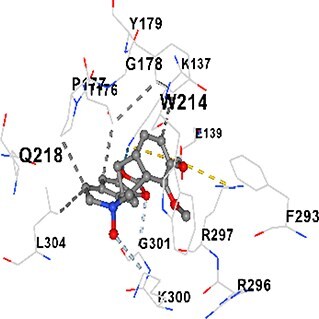
	PMCA (ATP2B1)	−8.0	Chain A: THR477 THR545 GLU546 PHE578 LYS583 SER584 MET585 LYS601 GLY602 ALA603 SER604 GLU605 ILE606 VAL633 LYS634 ILE637 GLU638 GLU643 ARG646 THR647 ILE648 GLY709 ASP710 ASN711 ILE712 ASN713 THR714 ARG716 ARG767	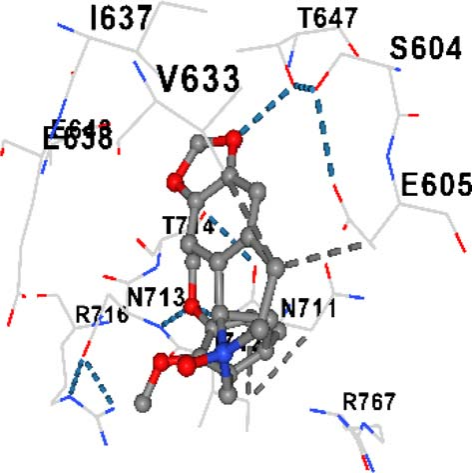
	SERCA (ATP2A1)	−8.6	Chain A: LEU52 GLU55 GLN56 ASP59 VAL62 LEU98 ASN101 ALA102 VAL104 GLY105 VAL106 GLN108 GLU109 GLU113 VAL304 ALA305 ALA306 ILE307 PRO308 GLU309 GLY310 LEU311 PRO312 ALA313 VAL314 PHE760 LEU764 ASN768 ASN796 ASP800 GLY801 ALA804 THR805	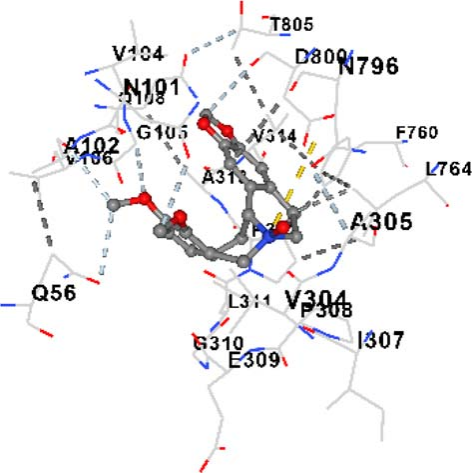
	RyR1	−6.4	Chain A: ARG2738 PRO2739 VAL2740 GLU2741 THR2742 LEU2743 ILE2804 TRP2807 PRO2808 GLU2811 ASN2881 TYR2882 HIS2883 ASN2884 THR2885 TRP2886 ARG2888 LYS2889 GLN2892 ASP2919 LYS2922	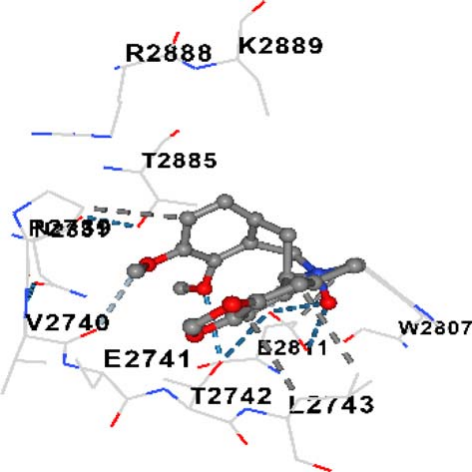
	IP3R (ITPR1)	−8.2	Chain C: ILE13 GLY30 LEU31 VAL32 ASP46 ASN47 PRO48 PRO49 LYS50 ARG53 LEU56 MET127 LYS128 SER129 ASN130 LYS131 ALA154 PHE224 MET225 HIS230 LYS236 GLY238 ASP239 VAL240 CYS254 ASP255 GLU256 ASN280 GLU286 PRO292 ARG305 HIS308 ALA310 THR311 GLY312 ASN313 TYR314 HIS359 GLY360 ASN361 VAL436 SER437 ILE439 ARG440 ASP441 ASP443 PHE444 ALA445 ASN446 SER449 SER450 PHE482 ASP485 VAL486 PRO487 ARG505 GLU511 GLN512 ASN513	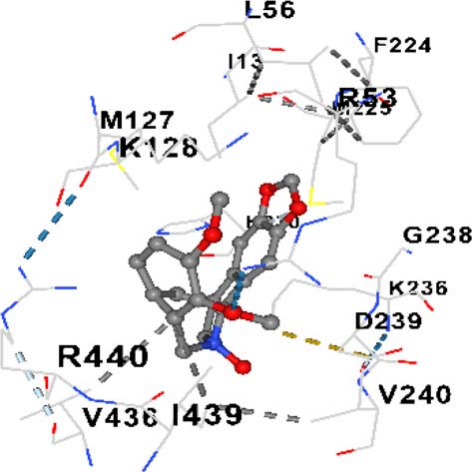

## Discussion

Calcium signaling is a delicately regulated mechanism that is of crucial importance for excitability of neuronal cells, plasticity of inter-axonal synapses, expression of certain genes, and longevity of cells.^27^^,^^28^ VGCCs, proteins with calcium binding capability, and certain calcium transporters collectively regulate calcium homeostasis within strict borders.^29^^,^^30^ Non-physiological conditions such as oxidative stress impair this delicate balance and cause over-accumulation of calcium ions in the cell, ultimately leading to neuronal injury or programmed cell death.^31^^,^^32^ This study assessed the protection provided by MAE against H₂O₂ -induced calcium imbalance in fPC12 cells.

According to the flow cytometry analyses, pretreatment with MAE resulted in a significant suppression of calcium ions influx into the fPC12 cells brought about by H₂O₂. This effect suggests that MAE revert calcium homeostasis to normal from its deranged status owing to the effects of oxidative stress. Accordingly, there occurred significant decrease in the expressions of a pair of L-type VGCC subtypes causing calcium influx into the cells, namely CACNA1C (Cav1.2 subunit) and CACNA1D (Cav1.3 subunit), by the effect of MAE. Excitotoxicity is a phenomenon tightly linked to these channels when they are overactive.^33^^,^^34^ MAE mitigates the intracellular calcium ions influx into overload by suppressing calcium entry into the cell via limiting the expression of these channels. This suppression was also evident by the observation that the CALM1 and CaMK2A levels were reduced. CALM1 stimulates certain targets including CaMK2A down the pathway via binding to Ca^2+^. Although those targets play important roles in neuroplasticity, their overactivity can be detrimental under the effects of oxidative stress.^15^^,^^35^ PMCA (ATP2B1) is a plasma membrane calcium ATPase, and SERCA (ATP2A1) is an endoplasmic reticulum calcium pump; both underwent modulation by the effect of MAE that allowed the latter to regulate calcium clearance. Both transporters are vital elements of calcium efflux from the intracellular free space into calcium stores, thereby serving calcium equilibrium.^36^^,^^37^ In addition, MAE caused a decrease in the levels of a pair of calcium channels, namely RyR1 and IP3R (ITPR1), which take part in calcium release from the ER.^38^^,^^39^ This raises the possibility that too much calcium release from intracellular stores is inhibited by MAE, a phenomenon that reinforces Ca^2+^ balance within a cell.

Each of the alkaloids that MAE contains displays different levels of biological function. Tetrahydropalmatine has been a focus of extensive research that has revealed that the alkaloid modulates dopaminergic pathways and acts as an antioxidant; it also enhances motor function and mitigates the effects of oxidative stress, thereby exerting neuroprotective activity in Parkinson’s disease models.^40–42^ This study found the evidence of tetrahydropalmatine having the most powerful binding affinity to a number of proteins involved in calcium handling, especially SERCA (ATP2A1) and IP3R (ITPR1), and its functional role in calcium homeostasis.

Allocryptopine is an isoquinoline alkaloid found in the chemical structure of Papaveraceae species; it reportedly exert antiarrhythmic, antispasmodic, and anti-inflammatory function.^43^^,^^44^ Despite the fact that less is known about its direct activity on calcium channels, our study provided the first evidence ever about its impact on VGCC expression and proteins involved in calcium regulation under oxidative stress.

Tetrahydroberberine N-oxide has a structure related to that of isoquinoline alkaloids, has been less studied from the aspect of its neuroprotective effects. Nevertheless, it is known that substances with properties similar to berberine activate AMPK pathways and show activity against oxidation and inflammation.^45–47^ Despite having the least powerful binding affinity of all the ligands studied in the present study, it is still likely relevant to calcium homeostasis because it interacts with SERCA (ATP2A1) and IP3R (ITPR1).

The isoquinoline core structure in tetrahydropalmatine, allocryptopine, and tetrahydroberberine N-oxide is reportedly the pivotal part of their biological activity and the main common property of the three compounds that makes it possible to regulate calcium homeostasis.^48^ This platform in their chemical structure allows them to enter the cells through the cell membrane and interact with targets within the cells that regulate calcium homeostasis. The methoxy (-OCH₃) groups are responsible for enhanced lipophilicity, which helps them to permeate through the membrane and bind to proteins of calcium regulation. Furthermore, these alkaloids contain protonatable amine groups confined to their ring systems, which make them possible for them to engage electrostatic interactions and make hydrogen bonds with target molecules including VGCCs. Particularly the N-oxide group of tetrahydroberberine N-oxide may rearrange the molecule’s electrons, change its redox behavior, and alter its interactions with proteins, e.g. SERCA and IP3R. Moreover, these alkaloids typically possess planar or semi-planar conformations that can improve their physical match with the surface of their target proteins, thus allowing more stable bonds. Altogether, these physical properties probably help these alkaloids mediate an effective regulation of calcium inflow, buffering, and storage, which culminates into improved calcium homeostasis under the effects of oxidative stress.

The analysis of the PPI network revealed a highly congregated and interwoven mesh of proteins taht are involved in calcium handling and targeted by MAE, which suggests a harmonized regulation of calcium homeostasis. The fact that the clustering coefficient was high and the *p* value of the enrichment factor hints at the plausibility of the biological importance of these interactions.

Molecular docking analyses revealed that MAE alkaloids exhibited high binding affinities with Ca^2+^ regulating target proteins. Binding scores below −8.0 kcal/mol in particular indicate strong ligand-protein interactions. In my analyses, it was observed that most of the MAE alkaloids bind to the active or allosteric sites of target proteins, and these bindings occur mostly through hydrophobic interactions, van der Waals forces, and in some cases hydrogen bonds. These data are a structural indicator of the inhibitory potential. These findings are also consistent with previous literature: Tetrandrine exhibits classical and allosteric inhibition effects by binding to the benzothiazepine binding site on Ca^2+^ channels and the ATP pocket of CaMKIIδ.^49^^,^^50^ Malbrancheamide derivatives bind to calmodulin like classical inhibitors and inhibit CaM function.^51^ Matrine and neferine bind to SERCA protein similarly to known inhibitors such as thapsigargin and cyclopiazonic acid, but act with different affinities.^52^^,^^53^ Similarly, in my study, MAE alkaloids bind to targets such as SERCA and CALM1 and interact with critical residues (e.g. GLU309, ASP59, GLU139, PHE89). These residues have been previously identified as regions where strong inhibitors bind. This supports that MAE alkaloids may exhibit direct inhibitory effects. In conclusion, molecular docking results, when evaluated in terms of both binding energies and contact residues, strongly suggest that MAE alkaloids act as potential inhibitors by binding to structural and functional regions of Ca^2+^ regulatory proteins. This coincides to a high degree with the effects of natural alkaloid inhibitors known in the literature and provides an important basis for the biological activity of MAE alkaloids.

Considering the known antioxidant properties of allocryptopine, tetrahydropalmatine and tetrahydroberberine N-oxide, it is clear that the effects of MAE on calcium dynamics may occur not only directly through interactions with calcium regulatory proteins but also indirectly through the reduction of ROS. The calcium regulation observed after MAE treatment is thought to be related to both the activation of antioxidant defense mechanisms and the decrease in the expression levels of calcium transporter proteins. This suggests that the effects occur through both direct molecular interactions and indirect mechanisms via the antioxidant response. Our previous studies have demonstrated that MAE exhibits strong antioxidant properties at various biochemical and cellular levels. In H₂O₂ and LPS-induced oxidative stress models, MAE has been shown to significantly reduce ROS levels, increase total antioxidant capacity, reduce oxidative stress index, and regulate the expression of antioxidant genes via NRF2-KEAP1 pathways (GCLC, HO-1, NQO1, and NRF2 ↑; KEAP1 ↓). In addition, its antioxidant effects have been confirmed through DPPH, hydroxyl radical, and superoxide anion scavenging activities, thiol/disulfide balance, and metal chelating capacities. MAE was observed to significantly increase the activities of major antioxidant enzymes such as SOD, CAT, and GPx. This suggests that MAE significantly contributes to the capacity to maintain intracellular redox balance. All these findings suggest that the effects of MAE on intracellular calcium dynamics may occur through both direct antioxidant mechanisms and indirect pathways related to the regulation of redox balance.^23–25^

Taking everything into consideration, the results of the present study put forward the notion that MAE has neuroprotective effects with multiple different aspects of activity including the modulation of calcium influx, buffering, storage, and mechanisms involved in signaling. This integral and coordinated calcium regulation under oxidative stress presents undeniable proof of the value of MAE alkaloids for use in the treatment of neurodegenerative diseases related to impaired calcium homeostasis. These findings are also consistent with previous studies demonstrating the neuroprotective, antigenotoxic, and acetylcholinesterase inhibitory effects of *G. corniculatum* (L.) RUD. subsp. *refractum* (Nábělek) Cullen (*G. grandiflorum* Boiss. & A. Huet subsp. *refractum* (Nábělek) Mory) extracts.^54–56^

## Conclusion

This study provided a study of potential neuroprotective effects of MAE with high allocryptopine, tetrahydropalmatine, and tetrahydroberberine N-oxide contents in an experimental model of oxidative stress on fPC12 cells. MAE led to significant reduction in intracellular calcium overload caused by H₂O, expression of the genes and proteins associated with the L-type calcium channel [CACNA1C (Cav1.2 subunit) and CACNA1D (Cav1.3 subunit)], and the expression of a number of crucial proteins involved in calcium handling, including CALM1, CaMK2A, PMCA (ATP2B1), SERCA (ATP2A1), RyR1, and IP3R (ITPR1). According to these results, one can argue that MAE mitigates oxidative stress-induced calcium handling in an effective manner and this action probably benefits its neuroprotective activity. Moreover, it was made clear by molecular docking analyses that the alkaloids of MAE showed strong binding affinity to protein targets associated with calcium signaling, especially SERCA (ATP2A1) and IP3R (ITPR1); this emphasized the fact that these compounds have the ability to exert direct modulatory activity on calcium homeostasis at the molecular level. The experimental and *in silico* findings as a whole showed that MAE and its alkaloids come forward as potential therapeutic agents for the prevention or treatment of neurodegenerative diseases related to calcium mishandling.

## Data Availability

The original contributions presented in the study are included in the article. Further inquiries can be directed to the corresponding author.
